# Editorial: Design, Synthesis, Characterization and Applications of Nanoclusters

**DOI:** 10.3389/fchem.2022.898480

**Published:** 2022-04-26

**Authors:** Laura Trapiella-Alfonso, Mariana Tasso, Gonzalo Ramírez García, Daniel Martín-Yerga, Antonio R. Montoro Bustos

**Affiliations:** ^1^ SEISAD team, Institute of Chemistry for Life and Health Sciences (i-CLeHS), UMR8060 CNRS, ChimieParisTech, Paris, France; ^2^ Departamento de Química, Facultad de Ciencias Exactas, Instituto de Investigaciones Fisicoquímicas Teóricas y Aplicadas (INIFTA), Universidad Nacional de La Plata—CONICET, La Plata, Argentina; ^3^ Biofunctional Nanomaterials Laboratory, Centro de Física Aplicada y Tecnología Avanzada, Universidad Nacional Autónoma de México, Querétaro, México; ^4^ Department of Chemistry, University of Warwick, Coventry, United Kingdom; ^5^ Chemical Sciences Division, Material Measurement Laboratory, National Institute of Standards and Technology, Gaithersburg, MD, United States

**Keywords:** synthesis, metal nanoclusters (NCs), bioapplications, optical properties, characterization

Metal nanoclusters (NCs) are nanocrystalline structures composed of tens of atoms that have attracted the attention of the scientific community in the last decades because of their strong photoluminescence and remarkable chemical properties. They represent the bridge between larger metal nanoparticles and organometallic compounds: due to a strong quantum confinement effect, NCs of sizes below 10 nm possess a distinctive molecule-like behavior upon interaction with an electromagnetic field, which is lost in larger nano-objects (Chakraborty and Pradeep, 2017). The first generation of metal NCs was formed by single noble metals such as gold, silver, platinum, copper, *etc*. (Lu and Chen, 2012) but nowadays the tendency in the field is oriented towards the development of bimetallic NCs or alloys to improve or have synergistic effects on their final optoelectronic and chemical properties. Furthermore, metal NCs exhibit size-tunable photoluminescence ranging from UV to the NIR, high photostability, two-photon absorption, and electroluminescent properties. These characteristics together with chemical ones have enabled the development of a wide panel of applications going from (bio)-sensing and catalysis to bioimaging and therapy (Du et al., 2020; Porret et al., 2020; Tang et al., 2020; Zare et al., 2021). In this research topic, by working with Au-Ag bimetallic NCs, B. Peng et al. demonstrated that the characteristic dual emission observed in this system depends on the pH and the degree of Ag substitution, so the system can be applied for ratiometric pH measurements.

A particularity, from a synthesis point of view, is that NCs need to be synthesized in the presence of a scaffold (ligands, polymers, biomolecules). These scaffolds also play a pivotal role in the optoelectronic properties of the obtained NC as they will modulate the final size and structure of the nanomaterial (Diez and Ras, 2011; Zare et al., 2021). Because of that, the origin of their photoluminescent properties has been unclear since its infancy, fostering thus dedicated research to elucidate these mechanisms. Mainly, two possibilities have been described that do not exclude each other. On one hand, the optoelectronic properties should be related to the intrinsic atomic energy levels of the metal core and thus governed by the quantum confinement. The second component that should be considered is the cluster’s surface where the properties will be governed by metal-ligand interactions (Yang et al., 2020). In this respect, throughout this Research Topic, the reader will find interesting works dedicated to shedding new light on the origin and modulation of the optical properties of bimetallic and metal alloy NCs. Peng et al. were able to establish the origin of the dual emission observed in Au-Ag bimetallic NCs and to attribute it to structural water molecules (SW) found at the interface between the NC surface and the ligand, thereby solving the dilemma between quantum size confinement effect and ligand-related surface mechanisms in this type of metal NCs. Using 1-dodecanethiol as ligand, dual emission at 440 and 630 nm could be observed and assigned to a single emitter, SW, presenting varying binding strengths with surface Au(I)- and Ag(I)- thiolate motifs. Furthermore, by tuning the amount of copper surface doping in [Au_9_Ag_12_(SAdm)_4_ (Dppm)_6_Cl_6_](SbF_6_)_3_ bimetallic NCs to form a trimetallic one [Au_9_Ag_8_Cu_4_(SAdm)_4_ (Dppm)_6_Cl_6_](SbF_6_)_3_, Deng et al. concluded that Cu dopants replace the Ag position in the peripheral DppmAg_2_Cl_2_(SR)_2_ structures and are crucial for explaining the changes in the optical properties of the parent Au_9_Ag_12_ NC, thereby highlighting the role of surface properties in the final optoelectronic behavior. However, since Cu oxidizes quickly, this trimetallic NC had time-dependent properties that fade over time under oxidizing environments while its parent bimetallic NC is unaffected.

Particularly, metal NCs are well suited for *in vivo* biomedical applications owing to their high biocompatibility (non-toxic metal cores and biocompatible surface chemistries), anti-fouling properties stemming from selected ligands allowing good circulation in the body, and ultrasmall sizes enabling a renal clearance pathway. In this special Research Topic, the use of Au NCs as fluorescence imaging contrast agents is exposed, as well as the use of this nanomaterial as a drug delivery system. Regarding the former, Hada et al. prepared bovine serum albumin (BSA)-stabilized gold NCs and carefully investigated their photoluminescence properties through fluorescence lifetime imaging microscopy (FLIM) under one and two-photon excitations, as well as via fluorescence spectroscopy. The nanostructures were characterized by photoluminescence in the first biological window, high photostability under continuous irradiation, and low photobleaching, which, together with their effective detection in agarose phantoms mimicking tissues, confirmed their aptitude to be employed as fluorescent nanotools in tissue bioimaging. Regarding their use as drug delivery vehicles, Wei et al. combined BSA-stabilized Au NCs with curcumin (a natural plant extract with poor water solubility and a regulatory effect on lipid metabolism) to form a new drug, NC-curcumin, with enhanced water solubility and therefore improved cell delivery potential. This nanosystem could effectively be endocytosed by H9c2 cardiomyocyte cells and employed to reduce intracellular lipid accumulation. Furthermore, the system had also an effect at reducing the increase in reactive oxygen species that occurs following lipid imbalances, therefore diminishing the risk of cell death.

While composed of a reduced number of contributions, this Research Topic addresses the key points in the NCs field and illustrates well the forefront and the trends of this promising group of nanomaterials. Therefore, we encourage the readers to explore it.
